# Triple Antiphospholipid (aPL) Antibodies Positivity Is Associated With Pregnancy Complications in aPL Carriers: A Multicenter Study on 62 Pregnancies

**DOI:** 10.3389/fimmu.2019.01948

**Published:** 2019-08-14

**Authors:** Maria-Grazia Lazzaroni, Micaela Fredi, Laura Andreoli, Cecilia Beatrice Chighizola, Teresa Del Ross, Maria Gerosa, Anna Kuzenko, Maria-Gabriella Raimondo, Andrea Lojacono, Francesca Ramazzotto, Sonia Zatti, Laura Trespidi, Pier-Luigi Meroni, Vittorio Pengo, Amelia Ruffatti, Angela Tincani

**Affiliations:** ^1^Department of Clinical and Experimental Sciences, University of Brescia, Brescia, Italy; ^2^Rheumatology and Clinical Immunology Unit, ASST Spedali Civili, Brescia, Italy; ^3^Immunorheumatology Research Laboratory, Istituto Auxologico Italiano, Milan, Italy; ^4^Rheumatology Unit, Istituto Auxologico Italiano, Milan, Italy; ^5^Rheumatology Unit, Department of Medicine-DIMED, University of Padova, Padova, Italy; ^6^Division of Clinical Rheumatology, ASST Pini-CTO, Milan, Italy; ^7^Department of Clinical Sciences and Community Health, University of Milan, Milan, Italy; ^8^Obstetrics and Gynaecology Unit, ASST Spedali Civili, Brescia, Italy; ^9^Obstetrics and Gynaecology Unit, IRCCS Ca' Granda Ospedale Maggiore Policlinico, Milan, Italy; ^10^Cardiology Clinic, Department of Cardiac Thoracic and Vascular Sciences, Thrombosis Centre, University of Padova, Padova, Italy; ^11^I.M. Sechenov First Moscow State Medical University, Moscow, Russia

**Keywords:** antiphospholipid (aPL) antibodies, pregnancy complication, adverse pregnancy outcomes (APO), thrombosis, anticardiolipin (aCL), lupus anticoagulant, anti-β2 glycoprotein I antibodies, antiphospholipid syndrome (APS)

## Abstract

**Objective:** Antiphospholipid antibodies (aPL) are risk factors for thrombosis and adverse pregnancy outcomes (APO). The management of the so called “aPL carriers” (subjects with aPL positivity without the clinical criteria manifestations of APS) is still undefined. This study aims at retrospectively evaluating the outcomes and the factors associated with APO and maternal complications in 62 pregnant aPL carriers.

**Methods:** Medical records of pregnant women regularly attending the Pregnancy Clinic of 3 Rheumatology centers from January 1994 to December 2015 were retrospectively evaluated. Patients with concomitant autoimmune diseases or other causes of pregnancy complications were excluded.

**Results:** An aPL-related event was recorded in 8 out of 62 patients (12.9%) during pregnancy: 2 thrombosis and 6 APO. At univariate analysis, factors associated with pregnancy complications were acquired risk factors (p:0.008), non-criteria aPL manifestations (p:0.024), lupus-like manifestations (p:0.013), and triple positive aPL profile (p:0.001). At multivariate analysis, only the association with a triple aPL profile was confirmed (p:0.01, OR 21.3, CI 95% 1.84–247). Patients with triple aPL positivity had a higher rate of pregnancy complications, despite they were more frequently receiving combined treatment of low dose aspirin (LDA) and low molecular weight heparin (LMWH) at prophylactic dose.

**Conclusion:** This study highlights the importance of risk stratification in pregnant aPL carriers, in terms of both immunologic and non-immunologic features. Combination treatment with LDA and LMWH did not prevent APO in some cases, especially in carriers of triple aPL positivity. Triple positive aPL carriers may deserve additional therapeutic strategies during pregnancy.

## Introduction

Antiphospholipid antibodies (aPL) are a heterogeneous group of autoantibodies reacting against phospholipids, phospholipid-protein complexes and phospholipid-binding proteins, detected by different assays: lupus anticoagulant (LA), anticardiolipin (aCL) and anti-β2 glycoprotein I (anti-β2GPI) antibodies.

The so called “aPL carriers” are individuals with persistent aPL positivity, in the absence of the clinical criteria of APS (pregnancy morbidity or thrombosis) (1). Sometimes aPL positivity is found in the presence of less specific features, defined as “non-criteria” manifestations (e.g., thrombocytopenia, valvulopathies, livaedo reticularis) (2) or the so called “lupus-like” manifestations; these patients are also included in the “aPL carriers” term.

The finding of the aPL positivity in a completely asymptomatic subject may happen during a laboratory work-up for non-autoimmune conditions. For example, aPL can be searched when a first-grade relative is affected by APS or as a second-level examination when a prolonged phospholipid-dependent coagulation time is found prior to surgery. Moreover, aPL are sometimes included in the diagnostic protocols to assess infertility causes performed before assisted reproduction procedures. Despite being asymptomatic, these subjects are theoretically at increased risk of vascular and obstetric events because they carry persistently positive aPL. In fact, aPL have been demonstrated to be pathogenic (3), therefore they should be considered as risk factors for thrombosis or pregnancy morbidity, with several other variables modulating the final clinical expression (4, 5).

The management of pregnant APS patients aims at preventing obstetric complications and maternal thrombotic events, tailoring a therapeutic strategy based on risk stratification. Risk stratification includes general factors (smoking, arterial hypertension, overweight, maternal age, diabetes mellitus, inherited thrombophilia) and disease-specific factors, particularly the so called “aPL profile.”

In most of the studies on APS patients, the “triple aPL profile” (3 positive aPL tests) is indicated as a major risk factor for thrombosis (6, 7) and pregnancy complications (8–12), while single aPL positivity seems to be generally associated with a favorable outcome (8).

Regarding the single tests, there is no definitive conclusion: some authors indicated LA as the strongest factor associated with obstetric complications (13), while others found that anti-β2GPI antibodies could be the most relevant ones (9, 14). Concerning the isotype, IgG is historically considered as more significant than IgM (15), but according to data from the European Registry of obstetric APS (EUROAPS), IgM isotype still remains important for the classification and the diagnosis of APS (16). Whether the same considerations could be valid also in patients with no history of aPL-related clinical events, has still to be demonstrated.

In patients with an established diagnosis of obstetric APS, combination therapy of low-dose aspirin (LDA) and low molecular weight heparin (LMWH) is regarded as the conventional treatment (17). At this moment, EULAR recommendations are available for pregnant aPL positive patients and can help clinicians in everyday practice, although being based mainly on expert opinion (18).

Regarding the specific subpopulation of pregnant women not fulfilling the APS criteria, and especially aPL carriers, LDA is usually part of the therapeutic strategy (19), even if the available studies failed to demonstrate its efficacy (20, 21). Possible limits of these studies seem to be mainly related to the heterogeneity of the patients included, regarding both the aPL profile or the associated risk factors. Importantly, the setting of aPL positivity in the context of an autoimmune disease should be considered as a completely different entity, as compared to the isolated aPL positivity in the absence of a definite autoimmune disease (22).

In a previous collaborative study, we retrospectively analyzed the pregnancy outcome of different clinical subgroups of aPL patients (11). Of note was that the outcome of aPL carriers was comparable to that of patients with a diagnosis of a definite thrombotic or obstetric APS.

Starting from these observations, the present study aimed at identifying factors associated with pregnancy complications in aPL carriers in a multicenter Italian cohort of prospectively followed pregnancies.

## Materials and Methods

### Study Cohort and Inclusion Criteria

Medical records of pregnant women regularly attending 3 Rheumatology centers with a Pregnancy Clinic from January 1994 to December 2015 were retrospectively evaluated.

The inclusion criteria were:

- Persistent aPL positivity;
- First prospectively followed pregnancy for each patient; if a patient had more than one prospectively-followed pregnancy, only the first was considered for the inclusion in the study.
- Singleton pregnancies.

### Exclusion Criteria

- Organ-specific or systemic autoimmune diseases (according to the international classification criteria), apart from autoimmune thyroiditis;
- History of thrombosis;
- History of complicated pregnancies;
- History of previous treated pregnancies (with or without complications);
- Pregnancies complicated by concomitant conditions (i.e., anatomical abnormalities, cervix dilatation);
- Pregnancies without any complications, but not prospectively followed in one of the 3 centers.

### Autoantibodies Detection

At the preconception visit or during the first trimester of pregnancy, a complete aPL profile composed by the three criteria assays, was available for each subject. The aPL were considered positive if confirmed at least 12 weeks apart. LA was detected by coagulation assay, according to the current guidelines of the International Society on Thrombosis and Hemostasis (23). The aCL IgG/IgM and anti-B2GPI IgG/IgM tests were performed with Enzyme-linked Immunosorbent assay (ELISA) technique, according to the current recommendations (24). The cut-off to determine positive aCL and anti-B2GPI was settled as the 99th percentile of healthy blood donors, and the levels of positivity were expressed as “low” and “medium-high titers” according to collaborative international consensus (25). “Non-criteria” aPL antibodies were not routinely tested.

Antinuclear antibodies (ANA), antibodies to extractable nuclear antigens (anti-ENA), anti–double-stranded DNA antibodies (anti-dsDNA) and complement C3 and C4 fractions were detected as for clinical practice. The aPL and the other immunological tests were performed in each of the three participating centers, by certified laboratories fulfilling European quality standards.

### “Non-criteria APS” and “Lupus-Like” Manifestations

The clinical charts of the patients were reviewed for the presence of “non-criteria APS” and “lupus-like” features as reported by the clinicians. According to a recent international consensus (2), “non-criteria APS” manifestations were defined as the occurrence of at least one of the followings: superficial venous thrombosis, thrombocytopenia, microangiopathy, heart valve disease, livedo reticularis, migraine, chorea, seizures and myelitis.

“Lupus-like” manifestations were defined as the presence of one of the typical features of Systemic Lupus Erythematosus (SLE), including arthralgia, Raynaud's phenomenon, photosensitivity and alopecia, in the absence of a diagnosis of SLE (26) or undifferentiated connective tissue disease.

### Acquired Risk Factors

Acquired risk factors were defined as one or more of the followings: obesity (body max index >30), current cigarette smoke, diabetes mellitus (fasting plasma glucose tests ≥126 mg/dl), dyslipidemia (total cholesterol blood levels ≥200 mg/dl and/or triglycerides >150 mg/dl), hyper-homocysteinemia (blood homocysteine ≥13 μmol/L), systemic arterial hypertension (≥140/90 mmHg), inherited thrombophilia [antithrombin or protein C or protein S deficiency, homozygous mutation of factor V Leiden or prothrombin or methylenetetrahydrofolate reductase mutation (MTHFR)].

### Pregnancy Complications

For the purpose of this study, a wider spectrum of complications was considered beyond the classical clinical manifestations as defined by international APS criteria (1). In particular, the occurrence of either maternal thrombotic events (venous and/or arterial) during pregnancy or the occurrence of adverse pregnancy outcomes (APO) related to aPL. APO were defined as one of the following events: spontaneous abortion (<10th weeks of gestation); fetal death (≥10th weeks of gestation); neonatal death before hospital discharge due to complications of prematurity; preterm delivery before 36th weeks of gestation with or without pre-eclampsia and HELLP syndrome (Hemolysis, Elevated Liver Enzymes, Low Platelet Syndrome).

### Statistical Analysis

Continuous variables were reported as mean value (± standard deviation), whereas categorical variables as proportion and/or percentage. Mann-Whitney test for continuous variables and Fisher's exact test or Chi-square test for categorical variables were applied as appropriate. Logistic regression was applied for multivariate analysis. *P*-values <0.05 were considered significant and Odds Ratio (OR) with 95% Confidence Interval (95% CI) was indicated.

## Results

### Description of the Cohort

The Pregnancy Clinics' database of each center were reviewed and 62 women met the inclusion criteria during the period comprised between 1994 and 2015. These patients had 69 pregnancies during the study period, but only the first pregnancy followed at one of the 3 participating centers was considered for further analysis, according to the inclusion and exclusion criteria listed above. Therefore, we considered 62 pregnancies in 62 women. The number of patients enrolled for the 3 centers were *n* = 26 in Brescia, *n* = 29 in Padua, and *n* = 7 in Milano. The majority were Caucasian (*n* = 59, 95.2%) and the minority were African (*n* = 3, 4.8%). Autoimmune thyroiditis was present in 13 patients (21.7%). Nineteen patients (30.6%) had at least one acquired thrombophilic risk factor (12 current smoke, 3 hypercholesterolemia, 3 obesity, 3 hyper-homocysteinemia and 2 systemic arterial hypertension). While 14 had a single acquired risk factor, and 5 had 2 simultaneous risk factors, none had more than 2 acquired risk factors. Inherited thrombophilia was present in 5 of 36 patients in whom it was tested (13.9%).

Non-criteria manifestations were observed in 6 out of 62 patients (9.7%): 2 livedo reticularis, 1 livedo reticularis with neurologic manifestations (migraine, seizures), 1 thrombocytopenia, 1 pulmonary arterial hypertension and 1 migraine recovered with anticoagulants.

Lupus-like manifestations were present in 5 out of 62 women (8.1%): 5 cases of photosensitivity, in 2 patients associated with mild arthralgias. Among these 5 patients, 2 had positive ANA, while none had positive anti-dsDNA. Globally, ANA were positive in 11 patients (17.7%), anti-ENA in 2 (3.2%) (anti-Ro/SSA in both patients) and anti-dsDNA in none.

Complement levels (C3 and C4 fractions) were available in the preconception period or in the first trimester for 34 patients (55%) and were reduced in 11 cases (29%).

### Description of the Pregnancy Complications

Mean maternal age at conception was 31.9 ± 5.1 years. Two pregnancies were induced by assisted reproduction techniques, both with *in vitro* fertilization (male infertility in one case, female infertility in the other case). A synthetic description of these complicated pregnancies is reported in Table 1.

**Table 1:** Description of the 8 complicated pregnancies.

**Therapy**	**Calendar year**	**Start of therapy**	**Non-criteria APS manifestations**	**Acquired risk factors**	**Inherited thrombophilia**	**Lupus-like manifestations**	aPL Profile^*^	**Complications**
LDA+ pLMWH	2005	12 w	YES (livedo reticularis)	2	Protein S Deficiency	NO	Triple	Fetal death (14 w)
LDA+ pLMWH	2001	6 w+ 11 w	YES (livedo reticularis)	2	Not present	YES	Triple	PE and Neonatal death (25 w)
LDA+ tLMWH	2010	7 w	NO	0	Not present	NO	Single (aCL IgM low titer)	Popliteal vein thrombosis (7 w)
LDA+ pLMWH	2009	8 w	NO	1	Not present	NO	Triple	Ischemic stroke (37 w)
LDA	2006	Pre-conceptional	NO	1	Increased APCR	YES	Triple	Fetal death (22 w)
LDA	2002	6 w	NO	0	Not present	NO	Single (aCL IgG medium titer, IgM low titer)	Spontaneous abortion (9 w)
LDA	2003	9 w	NO	2	Not present	NO	Triple	PE (35 w)
NONE	2013	27 w (LDA+ pLMWH)	YES (livedo reticularis)	1	Not present	YES	Single (aCL IgG low titer)	PE/HELLP syndrome (32 w)

*w, weeks; aCL, anti-cardiolipin; APCR, Activated protein C resistance; HELLP, Hemolysis, Elevated Liver Enzymes, Low Platelet Syndrome; LDA, low-dose aspirin; PE, preeclampsia; pLMWH, low molecular weight heparin at prophylactic dosage; tLMWH, low molecular weight heparin at therapeutic dosage*.

* *Number of positive aPL tests*.

Eight pregnancy complications in 8 patients were observed: 2 thrombotic events (3%) and 6 APO (9%). Thrombotic events were one deep venous thrombosis (7th week) and one ischemic stroke (37th week). APO recorded were: 1 spontaneous abortion, 2 fetal deaths and 3 pre-term deliveries before the 34th week with pre-eclampsia. In one patient with a severe preterm delivery (25th week), a neonatal death occurred.

### Analysis According to the Immunologic Profile

In the preconception or first trimester complete aPL assays, the positivity for each test was distributed as follows:

- LA in 10 (16.1%) (9 in the context of triple aPL positivity, 1 in the context of double aPL positivity)
- aCL in 39 (62.9%), including 28 IgG and 23 IgM (45.2 and 37.1%)
- anti-B2GPI in 44 (71.0%), including 27 IgG (43.5%) and 27 IgM (43.5%).

A triple aPL positivity was present in 9 women (14.5%), double positivity in 13 (20.9%) and single positivity in 40 (64.5%). None of the single positive patients had isolated LA positivity.

In the triple aPL positive group, as compared to single plus double positive group (Table 2), there was a significantly higher frequency of APO (p:0.003), despite more frequently receiving the combination treatment of LDA and LMWH (p:0.001). This group had also an increased frequency of acquired risk factors (p:0.019) and non-criteria manifestations (p:0.035).

**Table 2:** Analysis according to the type of aPL profile: comparison between triple aPL positive patients and the pool of single and double aPL positive patients.

	**Single aPL positive (*n* = 40)**	**Double aPL positive (*n* = 13)**	**Triple aPL positive (*n* = 9)**	*p*-value^*^
**PATIENTS CHARACTERISTICS**				
Age ≥35 years	17/40 (42.5%)	8/13 (61.5%)	1/9 (11.1%)	0.070
Acquired risk factors^a^	10/40 (25.0%)	3/13 (23.1%)	6/9 (66.7%)	0.019
Inherited thrombophilia^b^	2/21 (9.5%)	1/7 (14.2%)	2/8 (25.0%)	0.305
Non-criteria aPL manifestations^c^	3/40 (7.5%)	0/13 (0%)	3/9 (33.3%)	0.035
Lupus-like manifestations^d^	2/40 (5.0%)	1/13 (7.7%)	2/9 (22.2%)	0.149
**PREGNANCY OUTCOME**				
Complicated pregnancy	3/40 (7.5%)	0/13 (0%)	5/9 (55.5%)	0.001
Thrombosis	1/40 (2.5%)	0/13 (0%)	1/9 (11.1%)	0.271
Adverse pregnancy outcome	2/40 (5.0%)	0/13 (0%)	4/9 (44.4%)	0.003
**TREATMENT DURING PREGNANCY**				
No treatment	14/40 (35.0%)	6/13 (46.1%)	0/9 (0%)	0.050
LDA^e^	25/40 (62.5%)	5/13 (38.5%)	4/9 (44.5%)	0.719
LDA+LMWH^f^	1/40 (2.5%)	2/13 (15.5%)	5/9 (55.5%)	0.001

* *p-value calculated comparing triple vs. single plus double aPL positive patients*.

a *Acquired risk factors: obesity; current smoke; any previous diagnosis of diabetes mellitus, dyslipidaemia, systemic arterial hypertension*.

b *Inherited thrombophilia: antithrombin or protein C or protein S deficiency, homozygous factor V Leiden or prothrombin or methylenetetrahydrofolate reductase (MTHFR) mutation*.

c *Non-criteria aPL manifestations: superficial venous thrombosis, thrombocytopenia, microangiopathy, heart valve disease, livedo reticularis, migraine, chorea, seizures and myelitis*.

d *Lupus-like manifestations: arthralgia, Raynaud's phenomenon, photosensitivity, alopecia*.

e *LDA, low dose aspirin*.

f *LMWH, low molecular weight heparin*.

Moreover, 3 of the 5 triple aPL positive patients taking the LDA plus LMWH, developed pregnancy complications.

### Analysis of the Factors Associated With Pregnancy Complications

The comparison of clinical and laboratory features between patients with and without complicated pregnancies is illustrated in Table 3. At the univariate analysis, acquired risk factors (p:0.008), non-criteria aPL manifestations (p:0.024), lupus-like manifestations (p:0.013), triple positive aPL profile (p:0.001), were found to be associated with pregnancy complications. The combination treatment was also significantly more frequent in complicated pregnancies (p:0.007).

**Table 3:** Comparison between complicated and non-complicated pregnancies: univariate and multivariate analysis.

	**Complicated pregnancies (*n* = 8)**	**Non-complicated pregnancies (*n* = 54)**	***p*-value (univariate analysis)**
Age (mean ± SD)	28.3 ± 5.8	32.4 ± 4.8	0.028^§^
Nulliparity	6/8 (75.0%)	43/54 (79.6%)	0.670
Acquired risk factors^a^	6/8 (75.0%)	13/54 (24.1%)	0.008^§^
Inherited thrombophilia^b^	2/8 (25.0%)	3/28 (10.7%)	0.305
Autoimmune thyroiditis	2/8 (25.0%)	11/54 (20.4%)	0.670
Non-criteria APS manifestations^c^	3/8 (37.5%)	3/54 (5.6%)	0.024^§^
Lupus-like manifestations^d^	3/8 (37.5%)	2/54 (3.7%)	0.013^§^
Single aPL positivity	3/8 (37.5%)	37/54 (68.5%)	0.119
LAC	0/3 (0%)	0/37 (0%)	
aCL IgG/IgM	3/3 (100%)	13/37 (35.1%)	
aB2GPI IgG/IgM	0/3 (0%)	24/37 (64.9%)	
Double aPL positivity	0/8 (0%)	13/54 (24.1%)	0.186
aCL + LAC	0/0 (0%)	1/12 (7.7%)	
aCL + aB2GPI	0/0 (0%)	12/13 (92.3%)	
Triple aPL positivity	5/8 (62.5%)	4/54 (7.4%)	0.001^§^^*^
No treatment	1/8 (12.5%)	19/54 (35.2%)	0.258
LDA^e^	3/8 (37.5%)	31/54 (57.4%)	0.450
LDA+LMWH^f^	4/8 (50.0%)	4/54 (7.4%)	0.007

a *Acquired risk factors: obesity; current smoke; any previous diagnosis of diabetes mellitus, dyslipidemia, systemic arterial hypertension*.

b *Inherited thrombophilia: antithrombin or protein C or protein S deficiency, homozygous factor V Leiden or prothrombin or methylenetetrahydrofolate reductase (MTHFR) mutation*.

c *Non-criteria aPL manifestations: superficial venous thrombosis, thrombocytopenia, microangiopathy, heart valve disease, livedo reticularis, migraine, chorea, seizures and myelitis*.

d *Lupus-like manifestations: arthralgia, Raynaud's phenomenon, photosensitivity, alopecia*.

e *LDA, low dose aspirin*.

f *LMWH, low molecular weight heparin*.

§ Variables considered at the multivariate analysis;

* *p-value (multivariate analysis): 0.01, OR (95% CI): 21.3 (1.84–247)*.

Triple aPL positivity was the only variable significantly associated with APO at the multivariate analysis (p:0.01, OR 21.3, CI 95% 1.84–247) (Table 3).

### Analysis According to the Type of Therapy

Twenty of the 62 patients (32.3%) received no treatment during pregnancy; 42 (67.7%) received LDA, in combination with LMWH in 8 cases (12.9%) (Table 4). In 36 out of 42 patients, LDA was started in the first trimester (85.7%), while in 6 (14.3%) in the pre-conception period. In 7 cases heparin was used at prophylactic dosage, while in one it was introduced at a therapeutic dosage because of a deep venous thrombosis occurred at the 7th gestational week. In 6 out of 7 patients, the prophylactic dosage was increased during the third trimester (a period in which the thrombophilic risk dramatically increases) or whether patient's weight was ≥70 Kg, according to local practice. Low dose prednisone (5 mg/day) was used in 2 patients (3.2%) for mild thrombocytopenia. No adverse events related to anti-thrombotic treatment were recorded; in particular, no major bleeding, no placental abruption or heparin-induced thrombocytopenia.

**Table 4:** Analysis according to the type of treatment received during pregnancy.

	**None** **(*n* = 20)**	**LDA^e^** **(*n* = 34)**	**LDA^e^ + LMWH^f^** **(*n* = 8)**
**PATIENTS CHARACTERISTICS**			
Age ≥ 35years	8/20 (40.0%)	16/34 (47.1%)	2/8 (25.0%)
Acquired risk factors^a^	5/20 (25.0%)	11/34 (32.4%)	3/8 (37.5%)
Inherited thrombophilia^b^	2/10 (20.0%)	2/19 (10.5%)	1/7 (14.3%)
Non-criteria aPL manifestations^c^	1/20 (5.0%)	3/34 (8.8%)	2/8 (25.0%)
Lupus-like manifestations^d^	2/20 (10.0%)	2/34 (5.9%)	1/8 (12.5%)
**aPL PROFILE**			
Single aPL positivity	14/20 (70.0%)	25/34 (73.5%)	1/8 (12.5%)
Double aPL positivity	6/20 (30.0%)	5/34 (14.7%)	2/8 (25.0%)
Triple aPL positivity	0/20 (0%)	4/34 (11.8%)	5/8 (62.5%)
**PREGNANCY OUTCOME**			
Complicated pregnancy	1/20 (5.0%)	3/34 (8.8%)	4/8 (50.0%)
Thrombosis	0/20 (0%)	0/34 (0%)	2/8 (25.0%)
Adverse pregnancy outcome	1/20 (5.0%)	3/34 (8.8%)	2/8 (25.0%)

a *Acquired risk factors: obesity; current smoke; any previous diagnosis of diabetes mellitus, dyslipidemia, systemic arterial hypertension*.

b *Inherited thrombophilia: antithrombin or protein C or protein S deficiency, homozygous factor V Leiden or prothrombin or methylenetetrahydrofolate reductase (MTHFR) mutation*.

c *Non-criteria aPL manifestations: superficial venous thrombosis, thrombocytopenia, microangiopathy, heart valve disease, livedo reticularis, migraine, chorea, seizures and myelitis*.

d *Lupus-like manifestations: arthralgia, Raynaud's phenomenon, photosensitivity, alopecia*.

e *LDA, low dose aspirin*.

f *LMWH, low molecular weight heparin*.

## Discussion

Antiphospholipid antibodies have a clear pathogenic role in APS and should be considered as risk factors for thrombosis and APO. Patients with persistently positive aPL without the clinical APS criteria, the so called “aPL carriers,” are increasingly recognized in different contexts, such as the “non-criteria” manifestations or activated partial thromboplastin time (aPTT) prolongation found before surgical procedures. The incidence of thrombotic and obstetric events and the therapeutic strategy to prevent them, especially during pregnancy, are still poorly defined.

In a previous collaborative study, we observed that the rate of APO was similar in aPL carriers and in patients with definite thrombotic or obstetric APS (11). Moreover, the treatment assigned in the aPL carriers patients was globally less intensive than in the other subgroups, probably based on the absence of clinical criteria.

In the present study, focused on aPL carriers women, triple aPL positivity emerged as the only independent factor associated with pregnancy complications. This profile was previously identified as a major risk factor for both thrombosis and APO in the long-term follow-up of patients with primary APS (7). In the large multicenter PREGNANTS cohort, triple aPL positivity was associated with a lower live birth rate and a higher incidence of IUGR, compared with double aPL positive and LA negative women (14). Conversely, the prospective PROMISSE study identified LA as the main predictor of APO in aPL women, independently from triple aPL positivity (27). In our cohort LA was present only in the context of triple or double aPL positivity, while none of the single aPL positive patients had isolated LA, therefore we cannot comment on this subgroup. However, it is known that isolated LA positivity might be misleading, as it is more frequently observed in patients with diseases different from APS (28). Moreover, isolated LA may define a distinct subgroup of patients positive for anti-prothrombin/phosphatidylserine (anti-PT/PS) (usually IgM isotype) at lower risk of thromboembolic events (29).

In the next future, the risk stratification of aPL carriers will probably include also the “non-criteria” aPL tests. In fact, anti-B2GPI domain I antibodies have been associated with late pregnancy morbidity and thrombosis and were more frequently detected in triple aPL positive patients (30). Moreover, anti-PT/PS have been identified as risk factors for IUGR and pre-eclampsia (31).

Interestingly, at the univariate analysis, maternal age was significantly lower in the group of complicated pregnancies, conversely to what expected in the general population. We can assume that the presence of aPL, together with other risk factors, was able to elicit pregnancy complications at a younger age in these women.

Regarding the therapeutic aspects, the combination of LDA plus LMWH is currently considered as the conventional strategy for APS pregnant women (18), while this approach is not routinely used in aPL carriers patients (20, 22, 32). In our cohort only 8 patients received this kind of therapy; 3 of them, all with triple aPL profile, developed pregnancy complications. Moreover, in the EUROAPS cohort the “non-criteria APS” patients less frequently received a treatment during pregnancy, compared to the “criteria APS” one, despite experiencing a similar rate of complications (33). Together, these data suggest that clinicians are more prone to prescribe a full treatment if in the context of a definite obstetric APS, giving much importance to a previous history of APO. Based on our study, the combination treatment may be considered as preventative treatment in those aPL carriers at higher risk of obstetric and maternal complications, particularly in those with triple aPL positivity. These women could be counseled to start LDA before conception, as it is current practice in APS patients (18). A joint management with the obstetrician is necessary for the adjustment of heparin dosage during pregnancy (increase in maternal body weight, signs of placental insufficiency, etc.). The multidisciplinary management before and during pregnancy should aim at tailoring the treatment and the intensity of surveillance, yielding to the minimization of the risk of fetal and maternal complications (18, 34).

Another therapeutic option could be the addition of an immunomodulatory treatment, such as hydroxychloroquine (HCQ). If benefits of HCQ in SLE pregnancy are well-established (18), they are now increasingly described also in APS pregnancy (35). Its beneficial effects have been demonstrated in pre-clinical studies on the placenta, particularly at the trophoblast level (36), and in retrospective clinical studies with a reduction of pregnancy complications in aPL positive patients with or without APS (37–39), including refractory APS (40). The ongoing clinical trials HYPATIA (HYdroxychloroquine to improve pregnancy outcome in women with AnTIphospholipid Antibodies) (41) and HYDROSAPL (42) will provide further insights on this topic.

Regarding the potential side effects of treatments, we did not observe any case of major bleeding or heparin-induced thrombocytopenia. Our results are in line with those of a recent multicenter retrospective study, in which the occurrence of a bleeding complication in a cohort of pregnant APS patients was not increased by the anti-thrombotic treatment, including the combination of LDA plus LMWH (43). Therefore, the risk of bleeding should not prevent the physician from offering a combination treatment of anti-thrombotic agents to aPL carriers.

Finally, aside from pregnancy, aPL carriers should be considered as a population at increased risk of thrombosis, as demonstrated in different studies (7, 44, 45). Considering data of the obstetric APS women in the APS ACTION cohort, acquired risk factors, non-criteria manifestations and younger age at pregnancy morbidity represented risk factors for the development of thrombosis (subsequently recorded in 63% of patients) (45). In the present study, the patients who experienced complications during pregnancy were significantly younger. Therefore, they should be regarded as patients at possible increased risk of developing subsequent thrombosis.

Our study has some limitations, that include the relatively small sample size and the retrospective nature, even if all the pregnancies included were prospectively followed. Moreover, autoimmune thyroiditis was frequently recorded and a common reason for which aPL tests were requested, but data on thyroid function was not systematically collected. Future analysis should also consider thyroid-stimulating hormone (TSH) value, especially considering the recent endocrinology studies, in which TSH values at the upper limit of normal seems to be associated with early pregnancy losses (46).

Complement fractions levels were not systematically assessed as well. The complement system may gain utility in pregnancy monitoring as it plays an important role in the pathogenesis of obstetric APS (47); several evidences support the link between variations in the levels of different complement fractions and pregnancy complications (48–50). This pathogenetic pathway should be adequately addressed in the future as a potential therapeutic target.

In conclusion, the analysis of pregnant aPL carriers allowed the identification of triple aPL profile as an independent factor associated with pregnancy complications. The risk stratification of pregnant aPL carriers should include the definition of aPL profile to offer the combination treatment of LDA and LMWH to the “high-risk” patients. The value of additional therapeutic strategies, such as HCQ, will be defined in the next future; at the moment they may be considered for aPL carriers patients with lupus-like and non-criteria manifestations.

## Data Availability

All datasets generated for this study are avaialble upon request.

## Ethics Statement

The study was performed according to the principles of the Declaration of Helsinki and was approved by all the Local Ethic Committees (approval number 1088 in the Promoting Center). All patients signed a written informed consent.

## Author Contributions

M-GL, LA, AR, P-LM, VP, and AT designed the study. M-GL organized the database. M-GL, MF, LA, CC, TD, MG, AK, M-GR, AL, FR, SZ, LT, AR, P-LM, VP, and AT recruited and evaluated the patients and fulfilled the database. M-GL, MF, LA, and AT wrote the manuscript. All authors contributed reviewed the manuscript draft, read and approved the submitted version.

### Conflict of Interest Statement

The authors declare that the research was conducted in the absence of any commercial or financial relationships that could be construed as a potential conflict of interest.
